# Genome Sequence of a Porcine Bocavirus Detected in Feces of Domestic Minks in China

**DOI:** 10.1128/genomeA.01170-17

**Published:** 2017-11-09

**Authors:** Yan Wang, Jing Zhao, Min Zheng, Zhijian Liu, Jiaqi Yuan, Jieji Zhao, Quan Shen, Zhaobin Fan, Lili Jiang, Shixing Yang

**Affiliations:** a School of Medicine, Jiangsu University, Zhenjiang, Jiangsu, China; b Department of Gynecology, Rizhao Maternity and Infant Health Hospital, Rizhao, Shandong, China; c Department of Pharmaceutical Science and Technology, Heze University, Heze, Shandong, China

## Abstract

We report here the genome sequence of porcine bocavirus strain PBov-JZ08, which was isolated from mink feces in China. Sequence analysis implied that PBov-JZ08 clustered with three porcine bocaviruses.

## GENOME ANNOUNCEMENT

Bocaviruses are small nonenveloped viruses having an icosahedral head and a single-stranded liner DNA genome of approximately 4.5 to 5.5 kb. Bocaviruses are detected in humans and various animals, such as pigs, cattle, bats, canines, cats, gorillas, and sea lions (1–6). Recently, it was discovered in minks in China (7). Here, we report the near-complete nucleotide sequence of one pig bocavirus, strain PBov-JZ08, which was isolated from mink stool samples in China.

Strain PBov-JZ08 was detected from a total of 30 mink stool samples that were collected at an animal breeding farm in Liaoning province in 2016. The original aim of the study was to detect mink bocavirus in domestic minks by PCR. However, we found that the DNA sequences shared 87% nucleotide similarity with porcine bocavirus strain PBoV-KU14 identified in South Korea. Then, the genome of the virus which was named PBov-JZ08 was amplified by PCR. Strain PBov-JZ08 was 5,256 nucleotides in length and possessed a base composition of 35.92% A, 22.58% G, 21.97% T, and 19.53% C. The NS1, NP1, VP1, and VP2 open reading frames comprised 1,953 bp (positions 366 to 2,318 bp), 600 bp (positions 2,502 to 3,101 bp), and 1,851 bp (positions 3,146 to 4,996 bp), respectively. NS1 shared the highest amino acid (aa) identity of 87.9 to 90.6% with strains PBoV-H18 and PBoV-KU14. However, the length of NS1 was 15 aa longer than PBoV-H18 and PBoV-KU14, which could be attributed to a low conservation area of the longer 3′ terminal region of the NS1 gene (positions 2,240 to 2,318 bp, positions 626 to 650 aa). An additional G at the nucleoside position 2248 led to the later termination of NS1 compared to PBoV-H18 and PBoV-KU14. Like other porcine bocaviruses, NS1 contained conserved motifs associated with rolling circle replication, helicase, and the ATP-binding Walker loop motif (^424^GPASTGKT^431^). NP1 was the shortest among porcine bocaviruses with 199 aa, as PBoV-KU14. VP1 contained a putative conserved C-terminal motif (^51^QQRSFYFARQAQGAKRAR^68^) (8). The VP2 gene completely overlapped with VP1. The start codon was located 204 bp downstream of VP1, encoding 548 aa, of which a glycine-rich sequence (21 to 32 aa) was present in the N terminus.

The relationship between PBov-JZ08 and the other members of the *Bocaparvovirus* genus was determined by phylogenetic analysis based on the NS1 aa sequences. It showed that PBov-JZ08 clustered with three porcine bocaviruses, i.e., PBoV-SX (GenBank accession number HQ223038), PBoV-H18 (GenBank accession number HQ291308), and PBoV-KU14 (GenBank accession number KJ622366) to form a clade. Aligning NS1, VP1, VP2, and NP1 of PBov-JZ08 against human, porcine, bovine, feline, canine, and sea lion bocaviruses, the aa sequence identities were 33.4 to 90.6%, 33.6 to 91.8%, 38.6 to 92.4%, and 35.7 to 87.4%, respectively.

### Accession number(s).

The genome sequence of PBov-JZ08 was deposited in GenBank under the accession number MF085373.
